# Hydration Dynamics of Model Peptides with Different Hydrophobic Character

**DOI:** 10.3390/life12040572

**Published:** 2022-04-12

**Authors:** Laura Lupi, Brenda Bracco, Paola Sassi, Silvia Corezzi, Assunta Morresi, Daniele Fioretto, Lucia Comez, Marco Paolantoni

**Affiliations:** 1 Dipartimento di Matematica e Fisica, Università Roma Tre, 00146 Rome, Italy; laura.lupi@uniroma3.it; 2 Dipartimento di Chimica, Biologia e Biotecnologie, Università degli Studi di Perugia, 06123 Perugia, Italy; brenda.bracco@studenti.unipg.it (B.B.); paola.sassi@unipg.it (P.S.); assunta.morresi@unipg.it (A.M.); 3 Dipartimento di Fisica e Geologia, Università degli Studi di Perugia, 06123 Perugia, Italy; silvia.corezzi@unipg.it (S.C.); daniele.fioretto@unipg.it (D.F.); 4 IOM-CNR c/o Department of Physics and Geology, Università degli Studi di Perugia, 060123 Perugia, Italy

**Keywords:** hydration shell, solvation dynamics, depolarized Rayleigh scattering, ATR-FTIR, protein hydration

## Abstract

The multi-scale dynamics of aqueous solutions of the hydrophilic peptide ***N***-acetyl-glycine-methylamide (NAGMA) have been investigated through extended frequency-range depolarized light scattering (EDLS), which enables the broad-band detection of collective polarizability anisotropy fluctuations. The results have been compared to those obtained for ***N***-acetyl-leucinemethylamide (NALMA), an amphiphilic peptide which shares with NAGMA the same polar backbone, but also contains an apolar group. Our study indicates that the two model peptides induce similar effects on the fast translational dynamics of surrounding water. Both systems slow down the mobility of solvating water molecules by a factor 6–8, with respect to the bulk. Moreover, the two peptides cause a comparable far-reaching spatial perturbation extending to more than two hydration layers in diluted conditions. The observed concentration dependence of the hydration number is explained considering the random superposition of different hydration shells, while no indication of solute aggregation phenomena has been found. The results indicate that the effect on the dynamics of water solvating the amphiphilic peptide is dominated by the hydrophilic backbone. The minor impact of the hydrophobic moiety on hydration features is consistent with structural findings derived by Fourier transform infrared (FTIR) measurements, performed in attenuated total reflectance (ATR) configuration. Additionally, we give evidence that, for both systems, the relaxation mode in the GHz frequency range probed by EDLS is related to solute rotational dynamics. The rotation of NALMA occurs at higher timescales, with respect to the rotation of NAGMA; both processes are significantly slower than the structural dynamics of hydration water, suggesting that solute and solvent motions are uncoupled. Finally, our results do not indicate the presence of super-slow water (relaxation times in the order of tens of picoseconds) around the peptides investigated.

## 1. Introduction

Water which surrounds complex biomolecules, such as proteins, is expected to affect their structure, dynamics, and activity [1,2]. At the same time, water itself is deeply influenced by the presence of macromolecules that induce modifications on its molecular organization and mobility, as compared to the bulk [3,4]. Despite the huge number of theoretical and experimental investigations, the spatial extension of the perturbation around biomolecules and the extent of dynamical changes of the surrounding water are still a matter of intense debate [5,6,7,8,9,10]. The chemical and topological properties of the protein surface act conjunctly in modulating the structure and mobility of interfacial water. Possible couplings between the protein’s internal motions and the hydration dynamics could also be relevant in this matter [11,12], where a crucial role is played by the H-bond reorganization of water. This latter is itself a cooperative process that involves translational and rotational degrees of freedom, and takes place over a range of spatial and temporal scales [8]. Given the complex nature of a protein surface and dynamics, small model systems are frequently used to obtain fundamental insights on the biomolecular solvation features, as well as on the role played by specific molecular units or sequences [13,14,15,16].

An example is offered by simple peptides, such as ***N***-acetyl-glycine-methylamide (NAGMA) and ***N***-acetyl-leucinemethylamide (NALMA), the systems here investigated. NAGMA is considered a model for a hydrophilic H-bonding backbone, while NALMA, which also includes an extended hydrophobic side chain, is used as a model of amphiphilic species [17,18,19]. Several literature works on NAGMA and NALMA concern hydrated powders and highly concentrated solutions [20,21,22,23]. Under these crowded conditions, the presence of the hydrophobic molecular portion is found to impact the hydration properties of the peptide. Other studies have considered the hydration dynamics of NALMA and/or NAGMA under more diluted conditions, in which multiple hydration layers form around the solute. In this case, the resulting picture appears more ambiguous, and the role played by the hydrophobic portion (if any) does not clearly emerge [17,18,19,24,25,26]. Indeed, no substantial differences on the hydration dynamics of NALMA and NAGMA have been evidenced through neutron scattering (NS) experiments under diluted conditions [18]. Concerning the single molecule rotational motion, large retardation factors ***ξ***~10 (where ***ξ*** is the ratio between hydration and bulk water relaxation times) have been deduced by these investigations [18]. On the other hand, a rather weak dynamical perturbation (***ξ***~1.5), confined within the first hydration layer, has been derived by NMR experiments [24], in agreement with the prediction of the extended jump model (EJM) for water reorientation [3,4,27]. Moreover, THz experiments, exploring very fast collective modes, suggest that water molecules localized up to more than three hydration layers away from the peptides are dynamically affected [25], and noticeable solvation differences between NAGMA and NALMA have been evidenced, even under rather diluted conditions. Variations in hydration properties around polar and apolar groups have been also detected by THz spectroscopy for amino acids [28] and other model peptides [29]. 

Extended frequency-range depolarized light scattering (EDLS) experiments, which probe the structural relaxation of water on picosecond timescales, suggest that NALMA induces a considerable retardation (***ξ***~6–8) on surrounding water, which extends beyond the first two hydration layers [30,31]. The observed process relates to the rapid fluctuations of the collective anisotropy polarizability, and is mainly ascribed to the local (H-bond) reorganization of water involving changes of intermolecular distances (i.e., density fluctuations) [32,33,34,35]. These experiments have also evidenced that the hydration shells tend to overlap at increasing NALMA concentrations. This behavior has been ascribed to random contacts among solute particles, and not to the occurrence of aggregation phenomena [31]. 

EDLS operates in the frequency range from fractions to tens of thousands of GHz, and can provide a wealth of information on the molecular dynamics in aqueous solutions [9]. However, there remain some controversies on the interpretation of the complex spectral profile obtained by EDLS and by time-resolved optical Kerr effect (TR-OKE), its time-domain counterpart [35,36,37]. The issue concerning the existence of a spectral component attributed to super-slow solvating water, with relaxation times extending up to tens of picoseconds, appears particularly relevant [36,37]. 

In the present work, EDLS experiments have been performed on aqueous solutions of NAGMA, taken as a hydrophilic model peptide The results are compared to those derived by analogous experiments performed on aqueous solutions of the amphiphilic NALMA [31]. The main goal of the present work is to clarify if, and to what extent, solute-induced modifications of hydration water are related to the chemical character of the peptide. The results confirm the capability of EDLS to provide quantitative information on both the rotational motion of the solute and the fast dynamics of hydration water. Moreover, the study provides novel insights into the role played by the hydrophilic/hydrophobic molecular moieties in determining the solvation of amphiphilic peptides and their aggregation propensity in the water-rich domain. Additional information on the structuring of hydration water and peptide aggregation has been derived by Fourier transform infrared (FTIR) spectroscopy in an attenuated total reflectance (ATR) configuration. 

## 2. Experimental Section

**Figure 1:**
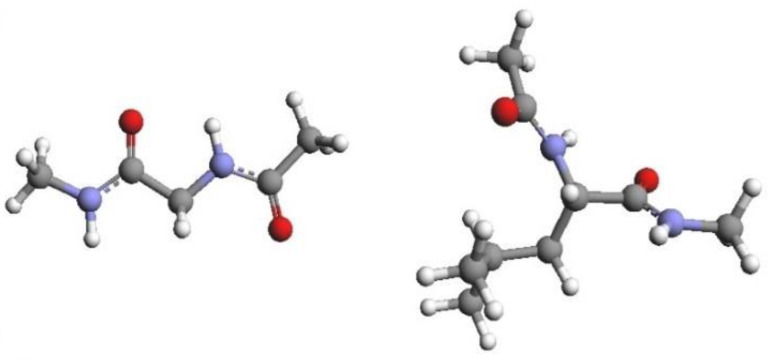
Graphical representation of the molecular structure of NAGMA (**left**) and NALMA (**right**).

The systems investigated are represented in Figure 1: NAGMA has a polar polypeptide backbone with CH_3_ end-caps (CH_3_–CO–NH–CH_2_–CO–NH–CH_3_); NALMA has the same backbone as NAGMA, but the glycine side chain is replaced with the hydrophobic leucine side chain ((CH_3_)_2_–CH–CH_2_). NAGMA was purchased from Bachem, with a purity higher than 99%. The solutions, prepared by weight using doubly distilled and deionized water, correspond to the concentrations of 25, 50, 75, 100 and 150 mg/mL of solvent. All of the samples were placed into a 10 mm path quartz cuvette after purification through cellulose 0.22 µm Millipore filters. EDLS spectra of aqueous NAGMA solutions were collected at T = 20 °C, over the spectral range spanning from 0.6 to 36,000 GHz, and compared with analogous data previously obtained for NALMA solutions [31]. In order to access such a broad frequency interval, a vertically polarized laser source was used, and the horizontally polarized scattered light was analyzed by means of two different spectrometers. The low-frequency region from 0.6 to 90 GHz was measured by means of a Sandercock-type (3 + 3)-pass tandem Fabry–Pérot interferometer, characterized by a finesse of approximately 100 and a contrast >5 × 10^10^. The higher frequency part of the spectra (60–36,000 GHz) was collected using a Jobin–Yvon U1000 double monochromator. Further details on the experimental setup are described in refs. [30,31]. Before joining the low and high-frequency part of the spectra, which overlap over approximately half a decade in frequency, the dark count contribution was subtracted from the raw intensity data. Finally, the imaginary part of the dynamic susceptibility, χ”, was calculated according to the relation χ”(ω) ∝ I(ω)/[n_B_(ω) + 1], where I(ω) is the measured depolarized intensity, and n_B_(ω) = 1/[exp(ω/k_B_T) − 1] is the Bose–Einstein occupation number. Additional NAGMA and NALMA solutions were prepared using both water and deuterated water as a solvent for the ATR-FTIR experiments. The spectra in the ATR mode were recorded with a FTIR Bruker spectrometer (mod. Alpha-P), equipped with a Platinum ATR module employing a single reflection (45°) diamond crystal. Each spectrum was obtained at room temperature, averaging over 120 scans at a resolution of 2 cm^−1^, using the spectrum of the empty ATR plate as the reference background. Spectral profiles in the frequency range 380–5000 cm^−1^ were converted using the “extend ATR correction” routine implemented in the Opus 7.5 Bruker Optics software, in order to obtain spectra comparable to those measured in the transmission mode. 

## 3. Results and Discussion

### 3.1. EDLS Data Analysis

**Figure 2:**
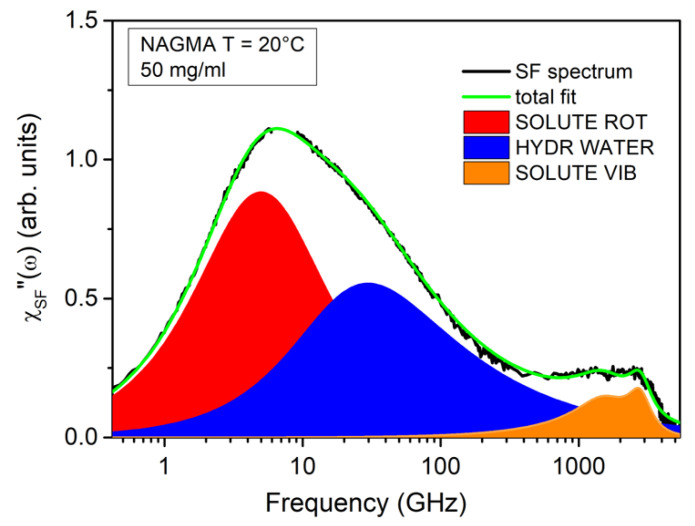
Solvent free (SF-green line) spectrum modeled as the sum of a Debye function (red filled area), a CD function with β = 0.6, (blue filled area), and two Brownian oscillators for the vibrational modes in the THz region (orange filled area).

EDLS experiments on aqueous solutions have been proven to be suitable for providing information on the rotational diffusion of the solute, the relaxation of hydration and bulk water, and the vibrational modes of the solute and solvent [9,30,31,37,38]. A full-spectrum analysis is needed to achieve quantitative information about all the processes involved. However, as a first step, the investigation of the solvent-free (SF) spectra provides extremely valuable hints for modeling the individual components of the spectra and for constraining several fitting parameters. The SF profile is obtained by the subtraction of the signal of the bulk solvent to the total EDLS spectrum. The subtraction is performed after normalization of the two spectra to the librational band of water [37], which is marginally affected by changes in temperature and concentration. Figure 2 shows, as an example, the result of the subtraction procedure for the NAGMA solution at a concentration of 50 mg/mL. The SF spectrum shows a multi-modal intensity excess due to the presence of different contributions in the frequency range from fractions to thousands of GHz. The most intense low-frequency peak, located at around 6–7 GHz, can be successfully reproduced by two components, as was already found in the case of NALMA solutions [31]. The first one is a symmetric Debye function, ***ℑm −*** {Δ_D_/[1 + iωτ_D_]}, where Δ_D_ and τ_D_ are respectively the amplitude and characteristic time of the relaxation process. This first component is peaked below 10 GHz, and can be attributed to the solute rotation [30,31]. The second, centered at around 30–40 GHz, can be assigned to the structural relaxation of hydrating water molecules. This contribution is modelled by an asymmetric Cole–Davidson (CD) function, ***ℑm −*** {Δ_CD_/[1 + iωτ_CD_]^βCD^}, where Δ_CD_, τ_CD_, and β_CD_ are the amplitude, characteristic relaxation time, and shape parameter, respectively. The average relaxation time can be obtained as <τ> = τ_CD_β_CD_. The same functional form can be used to reproduce the relaxation process in pure water, which peaks at around 270 GHz [30]. In both cases, the shape parameter has been fixed to β_CD_ = 0.6, in analogy to previous investigations [30,31,33]. The different frequency observed for hydration and bulk water reflects the degree of dynamical retardation induced by the solute on its proximal water [9,30,31,37,38]. Finally, the residual contribution in the THz region, which has also been identified in pure NAGMA powder [39], is due to the vibrational modes of the solute, and can be properly modeled by using two Brownian oscillator functions [30,31].

**Figure 3:**
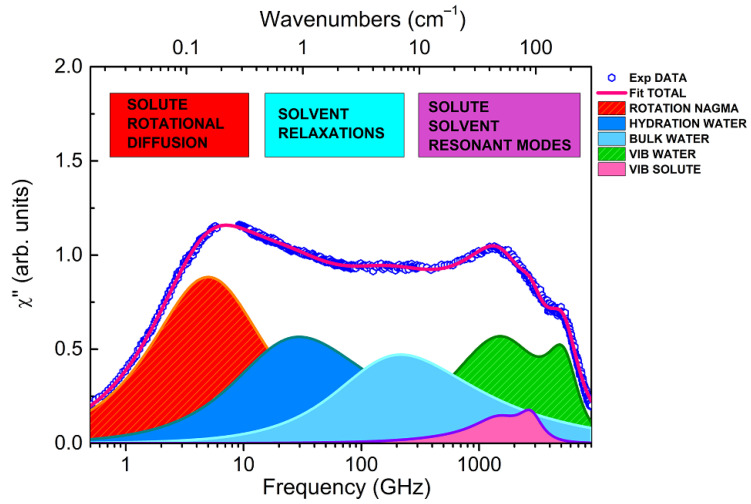
EDLS susceptibility data (symbols) of a representative NAGMA/water solution (50 mg/mL) at T = 20 °C. The total fitting curve and its decomposition into several contributions are indicated (see text). The small gap in the experimental data at around 7–8 GHz is due to the removal of a few spurious points arising from the leakage of the Brillouin peaks.

Taking advantage of this information, each experimental EDLS spectrum has been fitted in the entire frequency range by adding up the components evidenced by the SF spectrum with those present in the spectrum of pure water, as described in ref. [30]. These last components include one more relaxation term, and two damped harmonic oscillator (DHO) functions, accounting for two resonant contributions in the THz region, commonly assigned to the intermolecular bending and stretching modes of water [33]. An example of the fit result for a NAGMA solution is reported in Figure 3, where experimental data and best-fitting curves are shown. Disentangling specific solute-induced effects on the intermolecular modes of water in the THz region is a rather prohibitive task, and goes beyond the scope of the present study. On the other hand, the inclusion of these components is needed to properly model the relaxation region (extending below 1 THz) and to perform a consistent comparison with the results obtained by a similar spectral decomposition for NALMA solutions [31]. 

### 3.2. Water Dynamics

Based on the analysis described above, two relaxation components related to the solvent dynamics have been identified. This is consistent with what has previously been found in EDLS studies of aqueous solutions of solutes with different chemical nature, size, and complexity [9,38]. The contribution located at ~30 GHz is assigned to water molecules dynamically affected by the peptide, while the one located at ~200 GHz is assigned to water molecules with bulk-like dynamics, only marginally perturbed by the presence of solute. Notice that, since the parameter β_CD_ is the same for all the spectra, the resulting values of <τ> are all directly proportional to the values of either τ_CD_ or τ_max_ − this latter being τ_max_ = 1/(2πf_max_), with f_max_ the frequency of the maximum susceptibility. With β_CD_ = 0.6, as in our case, it is <τ> = 0.90τ_max_. The water relaxation process probed by EDLS is ascribed to local density fluctuations, in which the total anisotropic polarizability is modulated by changes in intermolecular distances (translations), mainly through dipole-induced dipole (DID) effects [34,35]. This structural relaxation relates to the local H-bond reorganization, and occurs quite rapidly in pure water, with an average relaxation time of 0.6–0.7 ps at room temperature [30,32,33]. This is considerably shorter than the average relaxation time for water reorientation dynamics (2–3 ps), as probed by NMR or fs-IR spectroscopy, and is widely discussed in the literature [3,4,24,27,40]. 

**Figure 4:**
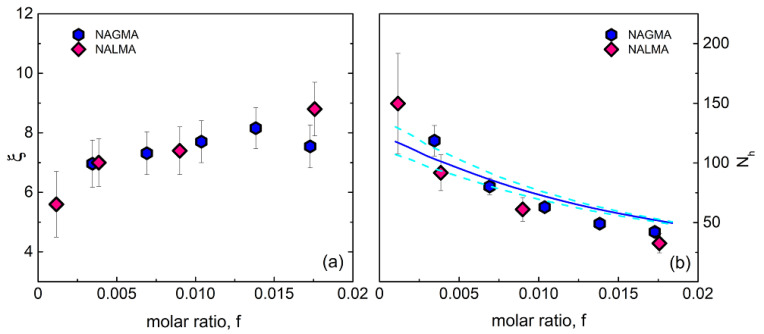
(**a**) Retardation ratio ***ξ*** = τ_hyd_/τ_bulk_ as a function of solute molar ratio for NAGMA and NALMA [31] solutions. (**b**) Average hydration number ***N_h_***, calculated as described in the text, as a function of solute molar ratio for NAGMA and NALMA [31] solutions. The values obtained from the water-sharing numerical model for non-interacting molecules (see text) are also represented (solid line: ***h*** = 6.4 Å; dashed lines: ***h*** = 6.2 and 6.6 Å).

From the spectral analysis, two relevant parameters can be simultaneously obtained: the solute-induced dynamical retardation factor ***ξ***, given by the ratio between hydration and bulk water relaxation times, and the solute hydration number, ***N_h_***, which represents the average number of dynamically perturbed water molecules per solute. This is given by ***N_h_*** = Δ_hydr_(Δ_hydr_ + Δ_bulk_)^−1^***f***^−1^, where Δ_hydr_ and Δ_bulk_ are the hydration and bulk water relaxation amplitude, respectively, and ***f*** is the solute to water mole ratio. In Figure 4, we compare the ***ξ*** (left panel) and ***N_h_*** (right panel) values obtained for aqueous solutions of NAGMA (at 20 °C) and NALMA (at 25 °C) [31], as a function of concentration. 

The results show similar retardation factors for NAGMA and NALMA, with ***ξ*** in the range 6–8, and slightly dependent on the concentration. This value is systematically higher than that obtained for the small hydrophobes Tert-butanol (TBA) and Trimethylamine ***N***-oxide (TMAO) and sugars (mono and disaccharides), while it is close to that observed for the protein lysozyme [9,38]. Moreover, the hydration number ***N_h_*** is also similar for the two systems, showing the same solute decreasing behavior with concentration. The physical origin of the marked reduction of ***N_h_*** might be either due to the occurrence of solute aggregation or to the increased probability of hydration shell overlap in randomly mixed solutions [41,42]. A simple water-sharing numerical model that accounts for the random superposition of hydration shells, described in detail elsewhere [41], has been shown to reproduce satisfactorily the ***N_h_*** behavior of NALMA [31]. Here, we apply the same model to NAGMA aqueous solutions, by representing the NAGMA molecule as a sphere of radius 3.0 Å. The simulation considers approximately 27,000 water particles and a number of solute molecules according to the given mole ratio. The experimental values are best reproduced by calculating the number of water molecules that fall within a geometric shell of thickness ***h*** = 6.4 Å around at least one solute molecule, and then dividing this number of hydration molecules by the number of solute molecules in the sample. It is worthy of note that recent molecular dynamics (MD) simulations on the ***N***-acetyltryptophanamide (NATA) dipeptide evidence that the average water molecules localized within 6 Å of the tryptophan side chain rotate around 1.6 times slower than all water molecules in the system. The retardation increases by decreasing the distance from the solute [43], indicating dynamic inhomogeneity.

The result of our numerical analysis is represented in Figure 4 using a blue line. We note that, despite the use of a simple model, the experimental data are described quite accurately. This suggests that, for both systems, the dominant contribution to the decrease of ***N_h_*** originates from the random overlap of hydration shells among neighboring solute molecules, rather than from aggregation effects [31]. It should be noted that the average distance between the centers of solute molecules, as simply estimated based on the sample molarity, ranges from around 30 Å to 13 Å in the investigated concentration range. Thus, considering a shell of thickness ***h*** = 6.4 Å around solutes with radius of ~3.0 Å (NAGMA) and ~3.5 Å (NALMA) [31], a significant increase in shell overlapping is expected with the rise of concentration.

In the limit of infinite dilution, we find ***N_h_*** = 125 ± 15 for NAGMA and ***N_h_*** = 130 ± 20 for NALMA [31]. This result implies a relatively long-range effect of peptides on the surrounding water, extending beyond the first two hydration layers. We note that, according to MD simulations [24], the number of water molecules localized within the first hydration layer is ~33 for NAGMA and ~43 for NALMA, with an increment of ~10 units due to the hydrophobic moiety.

The evidence that the two peptides, characterized by the same hydrophilic portion but different degree of hydrophobicity, exhibit essentially the same hydration properties represents a new element of discussion. The comparison suggests that, in both cases, the hydrophilic portion is largely responsible for the modification of the translational dynamics of surrounding water, leading to strong retardation and long-range perturbation. It is likely that the large effect caused by the hydrophilic backbone overcomes possible changes due to the hydrophobic portion of NALMA. We remark that for other solutes, such as sugars or simple osmolytes (TBA, TMAO), the dynamical perturbation of surrounding water is more contained and limited to the first hydration shell [9,38]. The presence of amide polar groups with their H-bonding ability might be responsible for the different behavior. Interestingly, concerning the single molecule rotational dynamics of water, it has been argued that the apolar part is the one that mainly affects the hydration dynamics, while the effect induced by the amide groups can be neglected [24]. On the other hand, other studies suggest that the hydrophilic portions have a major influence on both the dynamics and thermodynamics of hydration water [28,44]. Part of the discrepancies might derive from the fact that the hydrophilic and hydrophobic groups can affect the collective and the single molecule dynamics of the surrounding water molecules in a different way, and could also constrain water translational and rotational motions differently. In fact, neutron scattering experiments suggest that the effect of the model peptides on the rotational dynamics is limited to the first solvation layer, but it extends to the second one for the translational diffusion [17,18]. Additionally, MD simulations show that a peptide can slow down the translational motion of nearby water molecules up to distances of 12–13 Å, while leaving their orientational dynamics rather unaltered [44]. Moreover, a recent neutron scattering and MD investigation of protein solvation indicates that, compared to bulk water, the translation of hydrating water molecules is significantly more slowed down than their rotational motion [45]. 

We must note that the interpretation itself of the EDLS spectrum is, to some extent, problematic, and MD simulations with polarizability modeling could make a considerable contribution to clarifying fundamental issues [34,35,46,47]. However, MD studies performed on NALMA solutions have suggested that the main contribution to the EDLS signal arises from the solute rotation and bulk-like water relaxation, and a specific contribution from hydration water is not expected [35]. Notably, a substantial orientational ordering around the peptide, involving 3–5 water layers, is predicted, but this would not be reflected in any significant dynamical perturbation. As a matter of fact, these calculations were not able to reproduce the intermediate relaxation component (20–30 GHz) emerging from EDLS spectra [30,31,35]. In this respect, the present experimental results on NAGMA support our previous findings on NALMA [31], clearly indicating the presence of an additional spectral contribution at ~30 GHz (Figure 3). We also note that this contribution can be hardly attributed to a solute-solvent cross term that, according to the MD results, should be located at significantly lower frequencies (5–6 GHz) [35]. This is further confirmed by the observation that the features of this intermediate component are independent from the size, anisotropy, and dynamics of the solute, as will be demonstrated below.

Concerning the perturbation length-scale, which is another debated issue, we remark that the hydration numbers we have obtained are calculated assuming that the scattering cross section of water molecules does not change significantly moving from the bulk to the hydration shell. The short-range perturbation deduced under this assumption for other systems (sugars, TBA, TMAO) is indeed in agreement with other evaluations [34,37,46,48]. Nevertheless, the validity of such an assumption when specific amide groups are involved needs to be further ascertained. Overall, the present experimental findings can prompt additional theoretical efforts to improve the capability of computing EDLS spectra of aqueous solutions. 

### 3.3. Solute Dynamics

The component which peaked at a few GHz (Figure 3) and is modeled by a Debye function, is attributed to the solute dynamics [30,31]. For this symmetric functional form, τ_D_ corresponds to τ_max_ = 1/(2πf_max_). The higher the NAGMA concentration, the more intense the contribution. Previous EDLS studies on NALMA solutions assign this relaxation process to the rotational diffusion of peptide molecules [30,31], in general agreement with simulation results [35]. Here, an analogous assignment is made for NAGMA. We note that the presence of a GHz component related to the solute rotational motion is rather common in EDLS spectra, having been observed in several systems, such as formamide [49], sugars [46,50,51], and small hydrophobes (TBA [48] and TMAO [37]). Recently, the origin of low-frequency components in the spectrum of polarizability fluctuations has been stirring up new interest. Indeed, the GHz component detected in TMAO solutions by TR-OKE (the time-domain counterpart of EDLS) has been related to super-slow water molecules, whose dynamics are up to 50 folds slower than the bulk [36], at odds with previous interpretations [37,52]. Additionally, based on TR-OKE experiments, it was argued that this strong retardation of hydration water can be a more general effect, also induced by other solutes [36]. 

**Figure 5:**
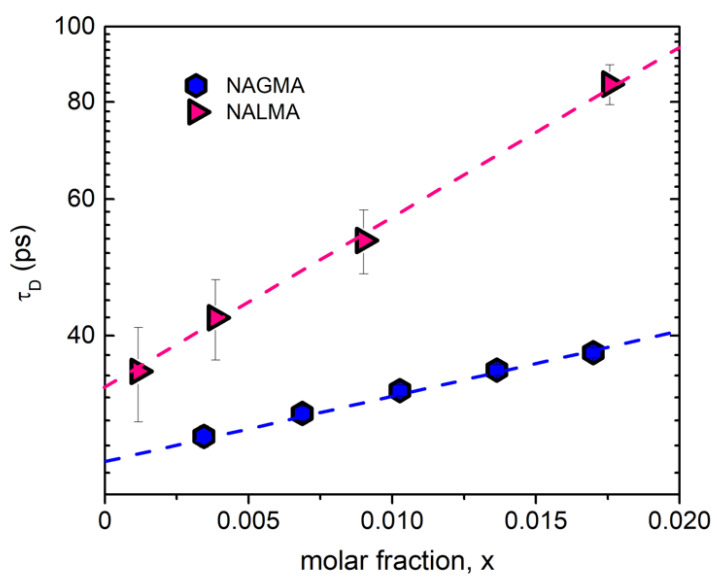
Rotational relaxation time (τ_D_) of NAGMA (this work) and NALMA [31] as a function of the solute mole fraction.

Figure 5 shows the concentration dependence of the relaxation time (τ_D_) associated with the low-frequency component detected in the EDLS spectra of NAGMA and NALMA solutions. In both cases, an exponential dependence on peptide mole fraction is evident, with the relaxation time of NALMA being systematically longer than that of NAGMA. For a single molecule rotational diffusion process (τ_sm_) derived by EDLS and OKE experiments, the relaxation time commonly follows the Stokes–Einstein–Debye (SED) relation, given by [49]:

τ_sm_ = *****V*****_h_*****η*****/(***k***_B_T) (1)

where ***k***_B_ is the Boltzmann constant, ***η*** is the shear viscosity of the solution, and ***V***_h_ the hydrodynamic volume of the solute molecule. With reference to the rotation of a spherical particle in a continuous medium (hydrodynamic limit) under stick boundary conditions, ***V***_h_ simply corresponds to the volume (***V***) of the rotating particle [49]. From the values of τ_D_ extrapolated at infinite dilution, when solute–solute correlations can be neglected (i.e., τ_D_ = τ_sm_), the hydrodynamic volume ***V***_h_ can be estimated from Equation (1), using the viscosity of neat water for ***η***. As a result, we obtain ***V***_h_ = 115 ± 20 Å^3^ for NAGMA and ***V***_h_ = 160 ± 30 Å^3^ for NALMA, which are in satisfactory agreement with their van der Waals volumes, ***V***_W_ = 125 Å^3^ (NAGMA) and ***V***_W_ = 192 Å^3^ (NALMA) [38]. This confirms the assignment of the GHz component to the reorientation of the peptide. It is important to note that the intensity of this component is higher for NAGMA, reflecting the greater anisotropic polarizability of this peptide. Overall, the present findings support the concept of a (rotational) contribution of the solute as the main origin of the GHz component detected in the EDLS spectra of the different systems previously investigated, which include solutes with molecular weight from ca. 75 to 350 Da [30,31,37,46,48,49,50,51]. We remark that the intermediate relaxation component (20–30 GHz) corresponds to a process with a relaxation time of 4–5 ps, independent of the peptide size and anisotropy. On the contrary, the slower rotational motion, whose characteristic time is one order of magnitude greater, differs significantly between NAGMA and NALMA. This further supports the idea that the intermediate component does not originate from a solute–solvent cross-correlation term [35].

The present analysis indicates that the reorientational mechanism of these peptides, under diluted condition, essentially consists of many small angular steps (small-step diffusion) that are likely driven by the reorganization of hydration water, occurring at a faster rate. The translational dynamics of water within the hydration shell of both peptides is retarded with respect to the bulk (<τ> values of 4–5 ps and 0.6 ps, respectively), but still considerably faster than the solute reorientation, suggesting that the two processes are dynamically uncoupled. Similarly, recent findings on the NATA dipeptide report that even if the reorientation dynamics of water slows down significantly close to the tryptophan moiety, it remains much faster than the reorientation of the indole group [43]. A different case is represented by the small molecule formamide, whose reorientation has been found to be fully coupled to that of the surrounding water [49]. We conclude that, in the case of peptides, the reorientational motion of the solute takes place within a relaxed local environment.

### 3.4. Intramolecular Vibrations

**Figure 6:**
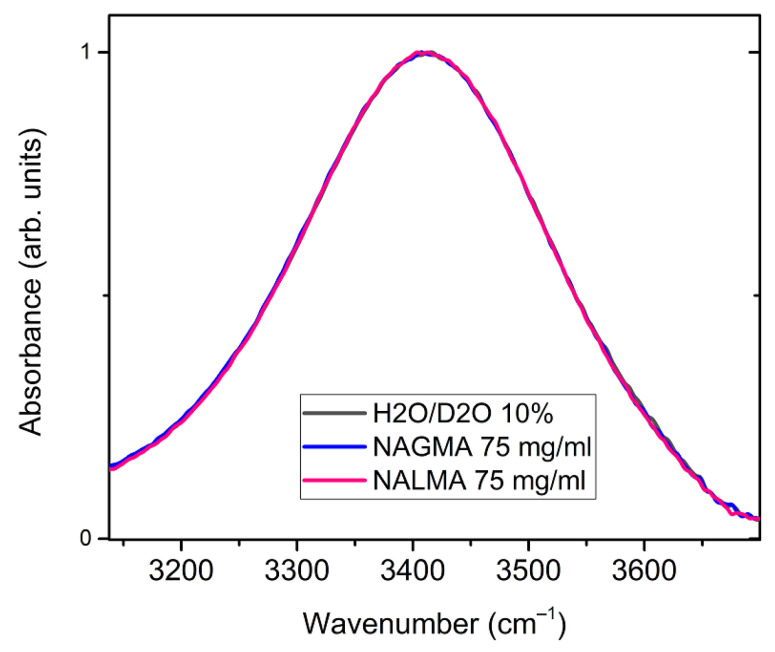
Normalized ATR-FTIR spectra of NALMA and NAGMA solutions (75 mg/mL), and of their solvent constituted by a H_2_O/D_2_O mixture (10% *w*/*w*). The spectrum is essentially due to the OH stretching modes of HOD species.

Structural information on hydration water can be obtained by analyzing the OH-stretching band of the infrared spectrum, which is sensitive to modulations of hydrogen bonding interactions [53,54,55,56,57]. Figure 6 shows a comparison between the ATR-FTIR OH-stretching bands obtained for NALMA and NAGMA solutions (75 mg/mL), and for the bulk solvent, composed by a mixture of H_2_O/D_2_O (10% *w*/*w*). The band of the solvent is mostly due to the OH stretching modes of HOD species, and is not influenced by intra- and inter-molecular vibrational coupling, thus facilitating the spectral interpretation [55]. The OH signal is sensitive to the strength of hydrogen bonds formed by the OH group (as proton donors): the formation of stronger H-bonds shifts the OH stretching distribution to lower frequencies (red-shift) [55,57,58]. 

No changes are observed between the solvent and the two solutions, therefore H-bonding modifications induced by the solute on surrounding water do not emerge under these conditions (x_NAGMA_ = 0.01 and x_NALMA_ = 0.007). Indeed, only minor changes have been recently evidenced by Panuszko et al. [59] between the spectrum of bulk HOD (OD stretching band) and the so-called NAGMA-affected spectrum, obtained by a differential analysis of concentration-dependent spectra. It was inferred by these authors that water around the peptide practically retains a structure similar to that of the bulk. Although the OH stretching band might not be sensitive enough to minor structural modifications under diluted conditions (Figure 6), we notice that, based on the analysis of EDLS spectra (Figure 4), at these concentrations, more than 50% of water molecules experience a strong dynamical perturbation. This complies with the idea that the fast dynamics of water is much more affected by a solute than its local structure, and highlights the ability of EDLS experiments to act as a sensitive probe of hydration properties.

**Figure 7:**
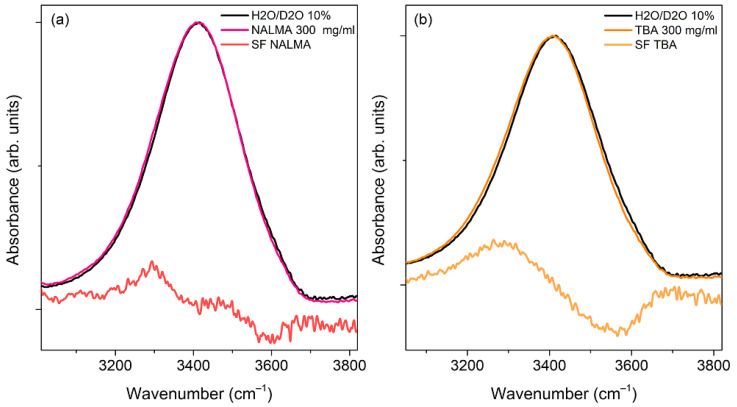
(**a**) Normalized ATR-FTIR spectra of a NALMA solution (300 mg/mL) and its solvent (H_2_O/D_2_O 10% *w*/*w*), together with the resulting difference spectrum (DS). (**b**) Normalized ATR-FTIR spectra of a Tert-butyl alcohol (TBA) solution (300 mg/mL) and its solvent (H_2_O/D_2_O 10% *w*/*w*), together with the resulting difference spectrum (DS). In both panels, DS spectra have been multiplied by a factor of 5 for visualization purposes.

To emphasize peptide-induced modifications, in Figure 7a the spectrum of a concentrated aqueous solution of NALMA (300 mg/mL; x_NALMA_ = 0.03) has been compared with that of the solvent. NALMA has been chosen for this analysis due to its higher solubility, with respect to NAGMA. At this concentration, minor spectral modifications start to appear, as emphasized by the resulting difference spectrum (DS), obtained by subtracting the spectrum of the solvent from that of the solution after normalization. Here, the minimum observed at ca. 3600 cm^−1^ might be related to the solute-induced small reduction of the population of weakly bonded OH groups of bulk water, while the peak at ca. 3300 cm^−1^ should be mainly attributed to the peptide NH stretching vibration. However, the difference is very subtle, even at this relatively high concentration. For the sake of comparison, Figure 7b shows the ATR-FTIR spectrum of an aqueous solution of Tert-butanol (300 mg/mL), together with that of the solvent and the corresponding DS spectrum. TBA is often used as a model to study hydrophobic hydration features, being that its solvent-exposed surface area is mostly due to the alkyl C(CH_3_)_3_ molecular portion [48,60,61]. Moreover, the alkyl moieties of TBA are similar in size to the hydrophobic leucine side chain (CH_3_)_2_CHCH_2_ of NALMA. The DS spectrum clearly shows that, when present at the same mass concentration, TBA causes much more apparent spectral changes than NALMA. We note that the effect on the water translational dynamics observed by EDLS for aqueous solutions of TBA was found to be significantly smaller than that of NALMA. The spectral modifications depicted in Figure 7b can be related to an increased population of stronger H-bonds formed by hydration water (hydrophobic hydration), as compared to the bulk [61]. Around the hydrophobic portion of TBA, water molecules partially modify their orientational order to maximize water–water H-bonds. On the other hand, the restructuring of water around NALMA molecules appears to be mainly driven by the hydrophilic molecular portion. In this respect, the intermolecular orientational order of bulk water seems to be largely maintained around the polar backbone, whose hydration should also involve the formation of direct water-peptide H-bonds [62].

Overall, the situation emphasizes the complex nature of the hydration phenomenon, and suggests that the local restructuring of water around NALMA is a rather collective effect, dominated by the local order imposed by its hydrophilic backbone. From a different perspective, it can be argued that the effect exerted on water by an apolar group decreases when the latter is close to a hydrophilic group, in line with the idea that the hydrophobicity of a free nonpolar group decreases when it is part of a peptide backbone [44].

**Figure 8:**
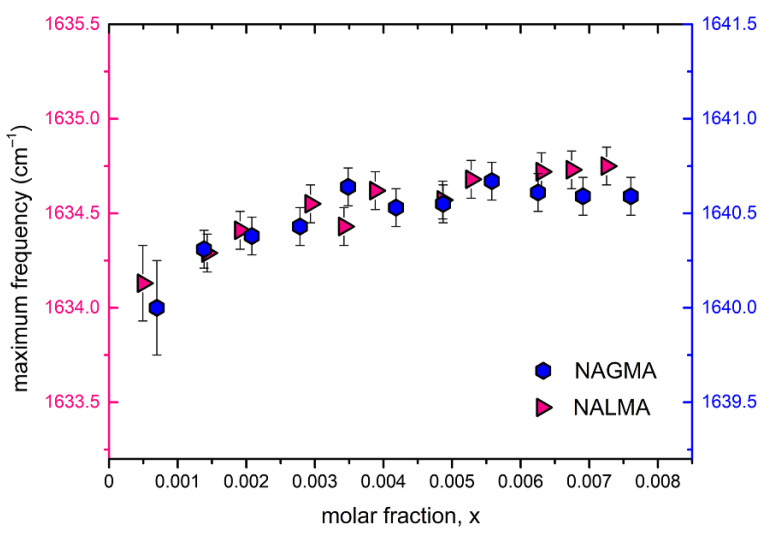
Amide I maximum peak position as a function of solute mole fraction.

Figure 8 shows the peak position of the amide I band (Am I) for both NAGMA and NALMA solutions as a function of concentration, in a range where EDLS experiments have evidenced relevant changes of the solute hydration numbers (Figure 4). Only a very small increase of the Am I position can be observed, indicating that structural changes around the amide groups are marginally reflected by these vibrations. A somewhat different situation has been pictured in a recent UV Raman study, in which a strong concentration dependence of the Am I band was evidenced under conditions of high dilution [63]. Our FTIR results suggest that the hydrophilic portion of both solutes remains fully hydrated in the investigated concentration range, and that the formation of peptide–peptide interactions is of minor relevance, consistently with the concept of a random distribution of solute molecules at all concentrations. Moreover, the data further confirm that the apolar group has a negligible impact on the hydration properties of the peptides.

## 4. Conclusions

In this work, the aqueous solutions of two model peptides have been investigated by EDLS spectroscopy in a broad frequency range from fractions of GHz to tens of THz. In particular. The results obtained for NAGMA, taken as a model hydrophilic peptide, have been compared to those obtained for NALMA, which contains both a hydrophilic backbone and a hydrophobic moiety. In agreement with previous investigations, the present study shows the presence of three district relaxation processes assigned to the structural translational dynamics of bulk-like (~200 GHz) and hydration water (~20–30 GHz), and to the reorientational dynamics of the solute (~4–6 GHz), with characteristic relaxation times of fractions, units, and tens of picoseconds, respectively.

As a main finding, our results reveal that NAGMA and NALMA affect the translational dynamics of surrounding water in a similar way. We estimate that, in both cases, the density fluctuations of hydration water are slowed down of a factor 6–8, as compared to bulk water. Moreover, the spatial region of dynamic perturbation extends for more than two layers at infinite dilution. Additionally, the observed reduction of the solute hydration number with solute concentration is explained by considering the random superposition of hydration shells, rather than aggregate formation. The comparison of the two peptide solutions indicates that the effect on the structural dynamics arises from the hydrophilic backbone, while the perturbation induced by the hydrophobic portion is negligible. Structural results derived by the analysis of ATR-FTIR spectra support this conclusion.

Being that the hydration features of peptides derived by EDLS are analogous to those observed for the protein lysozyme [64], it can be speculated that the translational dynamics of water around a protein is mainly determined by the hydrophilic portion of the exposed surface. Discrepancies in the literature on the extent of the dynamic perturbation might be partially explained by considering that water rotations are less affected than translations by a given solute [45].

Analysis of the solute relaxation contribution at GHz frequencies shows that the reorientation dynamics of NALMA is significantly slower than that of NAGMA at all concentrations. At an infinite dilution, the relaxation times agree with the prediction of the Stokes–Einstein–Debye equation, indicating that the peptide reorientation occurs through a small-step diffusion mechanism. It should be noted that the characteristic time of the translational relaxation process of hydration water molecules (4–5 ps) is of a similar magnitude as the orientational relaxation process detected by NMR [24]. Both processes are remarkably faster than the reorientation of the solute, suggesting that solute and solvent are dynamically uncoupled, and that water molecules do not follow the peptide during its motion. Finally, we find no indications of the presence of super-slow water molecules [36] around the model peptides here investigated.
